# Oxidative Stress, Antioxidant Defense, and Sleep–Wake Regulation in Psychiatric Disorders

**DOI:** 10.3390/antiox15050524

**Published:** 2026-04-22

**Authors:** Maurice M. Ohayon

**Affiliations:** Stanford Sleep Epidemiology Research Center (SSERC), School of Medicine, Stanford University, Stanford, CA 94305, USA; mohayon@stanford.edu

Sleep and wakefulness are fundamental biological functions that support adaptation, cognition, emotional regulation, and adaptive behavior. Yet disturbances in these functions are still too often treated as secondary features of psychiatric illness rather than as integral components of its biological architecture. This view is becoming increasingly difficult to defend. Across many psychiatric disorders, alterations in sleep and wakefulness may precede the full syndrome, worsen during symptomatic periods, persist during partial remission, and predict poorer outcomes or recurrence [1]. Sleep–wake dysregulation should therefore be considered as more than an associated symptom domain; it represents one of the clearest clinical windows into the mechanisms that shape resilience and vulnerability in the brain and body.

Oxidative stress and antioxidant defense occupy a central position within these mechanisms. Reactive oxygen species (ROS), particularly those generated through mitochondrial metabolism, are essential for cellular signaling, adaptation, and homeostatic regulation [2,3]. When ROS production exceeds buffering capacity, oxidative imbalance can alter signaling, amplify inflammation, and reduce energetic efficiency [2,3]. This balance is especially important in the nervous system, where energy demand is high and tolerance for instability is low.

The convergence of redox biology, antioxidant systems, and sleep–wake regulation is now difficult to ignore when considering psychiatric symptoms and long-term clinical course [3,4,5,6,7,8]. Sleep loss can alter oxidative regulation beyond the acute period of deprivation, and intermittent hypoxia may shape both somatic risk and psychiatric vulnerability. Antioxidant defenses, in turn, help maintain tissue integrity, network stability, and adaptive behavior. Symptoms such as hypersomnolence, fatigue, and non-restorative sleep may therefore reflect not only subjective complaints, but also reduced biological resilience [5,6,8,9,10,11].

A major problem in this field is conceptual. Oxidative stress is often treated as a downstream marker of damage, whereas sleep and wake disturbances are viewed as secondary consequences of primary disease processes. Both perspectives are too restrictive. Between normal physiology and overt pathology lies a broad zone of biological instability in which regulatory systems remain functional but operate with reduced efficiency and reserve. Compensation may still hold but at the cost of fluctuating performance, altered vigilance, mood dysregulation, and multi-symptom clustering [4,6,8,9,10].

This intermediate zone deserves greater attention because it is often where clinical observation and mechanism begin to meet. Patients do not usually present with obvious lesions, organ failure, or clear neurodegeneration. More often, they show fluctuating capacity: one day they function reasonably well, another day they show cognitive slowing, unstable alertness, reduced motivation, or poor tolerance to stress. These patterns are difficult to explain when psychiatry, sleep, and oxidative biology are kept in separate domains, and become far more intelligible once we accept that functional systems may become unstable long before structural damage is evident.

One way to understand this instability is through the convergence of multiple upstream stressors on mitochondrial function and redox regulation. Sleep deprivation or fragmentation, hypoxia, circadian desynchronization, inflammation, metabolic stress, aging, and chronic psychosocial burden can each increase mitochondrial load and alter ROS dynamics [3,4,5]. What follows depends on both the intensity of the challenge and the ability of antioxidant and buffering systems to preserve biological stability [12,13]. The key issue is not ROS in isolation, but the ability to regulate oxidative fluctuations while maintaining energetic efficiency, adaptive signaling, and network integrity. This is especially pertinent in psychiatry, where disorders rarely arise from a single insult and more often reflect the cumulative interaction of biological and environmental pressures over time [1,6,8].

This leads to the concept of mitochondrial resilience: the ability of cells and neural systems to maintain redox equilibrium, energetic efficiency, and functional stability under changing demand [2,12,13,14,15]. Such resilience likely varies across individuals and across systems. It may help explain why some people maintain stable wakefulness, cognition, motivation, and emotional regulation despite stress, while others develop persistent hypersomnolence, fatigue, cognitive slowing, affective instability, or impaired adaptive engagement [1,6,8,9,10,11]. In this framework, redox regulation becomes a shared biological dimension shaping symptom clustering, illness expression, and vulnerability across diagnostic boundaries.

Sleep–wake regulation provides a particularly revealing setting in which to examine these mechanisms. Wakefulness is metabolically demanding, requiring sustained neuronal firing, continuous integration of internal and external signals, dynamic motivational mobilization, and rapid adaptation to changing demands. Sleep contributes to restoration, recalibration, and stabilization of biological systems [3,4]. The sleep–wake cycle can therefore be understood as an oscillation between biological demand and biological recovery. Disruption of this oscillation through insufficient sleep, fragmentation, circadian perturbation, or hypoxic stress may signal impaired metabolic and redox regulation, rather than an isolated disturbance [3,4,5].

Patients do not simply say that they are sleepy or insomniac. They describe unstable daytime functioning, loss of mental endurance, reduced capacity to stay engaged, higher effort costs for ordinary cognitive tasks, and lower tolerance to internal or environmental challenge. Such descriptions suggest that the issue is not always sleep quantity alone. It may instead be the capacity to maintain stable adaptive functioning across the wake period. This observation does not reduce sleep disorders to mitochondrial biology, but it does suggest that sleep–wake complaints may reveal deeper regulatory disturbances than those captured by traditional symptom labels.

Intermittent hypoxic stress offers a clear example of repetitive oxidative challenge [5,16]. Fluctuations in oxygenation affect not only respiration and sleep continuity, but also mitochondrial function, inflammatory activity, redox balance, and longer-term tissue vulnerability [5,16]. Sleep medicine therefore offers a particularly valuable clinical and physiological arena in which to examine how patterned biological stress is translated into chronic dysfunction. This is highly relevant to psychiatric disorders, in which disturbances of sleep, wakefulness, cognition, and mood often coexist and in which symptom persistence suggests more than episodic disease activity [10,11,17,18].

A major challenge is to connect these biological processes to the heterogeneity of clinical phenotypes. In psychiatry, disturbed sleep may coexist with depression, anxiety, pain, cognitive complaints, metabolic dysregulation, or persistent fatigue. Hypersomnolence may appear alongside mood symptoms, reduced motivation, or cognitive slowing without fitting neatly into a single classical category [9,10,11]. In epidemiology, such constellations point to latent dimensions of vulnerability rather than sharply bounded disease entities. A redox-based resilience framework does not replace diagnostic distinctions, but it does help explain overlap, variability, and divergent trajectories [11,17].

Within the brain, this framework is especially relevant to neuro-modulatory systems governing arousal and adaptive engagement. Dopaminergic pathways contribute to salience and motivation, orexinergic systems stabilize wakefulness and integrate metabolic signals, and noradrenergic circuits sustain vigilance and stress responsiveness, thereby providing a natural bridge between sleep–wake regulation and biological resilience [19,20]. These systems are not only neurochemical; they are also bioenergetic. Their stability depends on mitochondrial integrity and redox balance. Under oxidative strain or inadequate buffering, their activity may become noisy, unstable, or inefficient. Clinically, this may be experienced not only as sleepiness, but also as unstable wakefulness, greater effort cost, reduced motivational energy, attentional collapse, cognitive slowing, and lower resilience under stress [19,20].

Antioxidant defense is central to this interpretation. Antioxidant systems should not be viewed simply as passive protection against oxidative excess. They are part of the regulatory architecture that allows biological systems to function under fluctuating demand. In this context, mitochondrial superoxide dismutase (SOD2) is of particular interest. As a major component of mitochondrial oxidative buffering, SOD2 can be viewed as an illustrative regulatory node within a broader resilience network [12,21]. Its significance lies not only in limiting oxidative injury, but also in preserving the conditions under which signaling remains adaptive, energetic efficiency is maintained, and neural activity does not shift toward instability. The point is not to reduce complex psychiatric phenotypes to a single antioxidant pathway. It is to recognize that SOD2-dependent buffering exemplifies how antioxidant defenses may influence whether stress exposure leads to adaptation, instability, or progressive dysfunction [12,13,21].

This framework supports dimensional and transdiagnostic thinking. Symptoms such as hypersomnolence, fatigue, cognitive inefficiency, reduced motivation, stress intolerance, and non-restorative sleep need not be treated as separate complaints awaiting separate explanations. They may instead represent different expressions of a reduced capacity to maintain stable adaptive function under physiological and psychosocial load. The same symptom may therefore have very different implications depending on the resilience of the system in which it appears [10,11,17].

This perspective has direct implications for research design. Mechanistic studies are needed to clarify how ROS production, antioxidant defenses, mitochondrial dynamics, and hypoxic stress influence neuronal and glial stability [12,13,21]. Physiological and translational studies can examine how sleep deprivation, circadian disruption, intermittent hypoxia, and metabolic strain alter redox equilibrium in vivo [3,4,5]. Clinical and epidemiological work can determine how these processes relate to insomnia, hypersomnolence, fatigue, mood symptoms, cognitive complaints, pain, multi-symptom clustering, and long-term psychiatric outcomes [1,8,10,11,17,18].

These research directions will be most productive if disciplinary boundaries are treated as provisional rather than fixed. Molecular findings need clinical translation, but clinical patterns also need biologically plausible organizing principles. Sleep research should be central here because it sits at the junction of physiology, behavior, and disease. Likewise, psychiatric research should continue to develop frameworks that connect subjective symptoms, system-level function, and biological regulation. A shared focus on oxidative stress, antioxidant defense, and sleep–wake instability could help define a genuinely integrative field of research. Ultimately, the importance of this topic lies in its potential to change how psychiatric vulnerability itself is understood. Sleep instability, hypersomnolence, fatigue, cognitive inefficiency, and mood dysregulation may reflect different expressions of an organism’s capacity, or reduced capacity, to maintain stable adaptive function under stress. In that sense, oxidative stress and antioxidant defense may define part of the biological grammar through which resilience and fragility are expressed across the lifespan.

We hope the contributions gathered here will help establish this field not as a marginal intersection, but as a central area of inquiry for contemporary psychiatric and sleep research.
